# Determination of Toxic Metals in Indian Smokeless Tobacco Products

**DOI:** 10.1100/tsw.2009.132

**Published:** 2009-10-14

**Authors:** Dhanashri Dhaware, Aditi Deshpande, R. N. Khandekar, Rohini Chowgule

**Affiliations:** Indian Institute of Environmental Medicine, Kasturba Hospital, Mumbai, India

**Keywords:** smokeless tobacco, metals, lead, cadmium, arsenic, copper, mercury

## Abstract

This study targets the lesser-known ingredients of smokeless tobacco products, i.e., the toxic metals, in Indian brands. The metals selected in the study included lead (Pb), cadmium (Cd), arsenic (As), copper (Cu), mercury (Hg), and selenium (Se). The differential pulse anodic stripping voltammetry (DPASV) technique was used for estimating the metals Pb, Cd, and Cu; square wave voltammetry for As; and the cold vapor atomic absorption technique for Hg. The resulting levels of the metals were compared to the daily consumption of the smokeless tobacco products. It was observed that almost 30% of gutkha brand samples exceeded the permissible levels of metals Pb and Cu, when compared to the provisional tolerable intake limits determined by the FAO/WHO. The reliability of data was assured by analyzing standard reference materials.

## INTRODUCTION

The tobacco plant (*Nicotiana tabacum*) is widely known for its leaves, which are smoked, chewed, or sniffed for various effects. It is well documented that the addiction of tobacco comes from the chemical nicotine[1], which is harmful to humans. Tobacco contains over 19 known carcinogens and at least 30 metallic compounds, comprising heavy metals[2,3,4]. Harmful effects on human health are associated with exposure to the heavy metals lead (Pb), cadmium (Cd), mercury (Hg), arsenic (As), selenium (Se), and nickel (Ni). These metals have been extensively studied[5] and their effects on human health have been regularly reviewed by international bodies, such as the World Health Organization (WHO). These heavy metals are found in the atmosphere as well as many man-made sources, and they do not have any metabolic function, as such, in the body[6].

In India, a very popular form of tobacco is the smokeless tobacco (commonly known as gutkha, zarda, khaini, and others) consisting of a mixture of tobacco and other constituents. In addition to nicotine, the major groups of carcinogens in smokeless tobacco products (STPs) include nonvolatile tobacco-specific nitrosamines (TSNA), *N*-nitrosamine acids, and other constituents. Reports of toxic metals in cigarettes[7,8] and tobacco[9] led us to study their presence and accumulation in smokeless tobacco variants[10]. The concentration of these metals in humans depends on the daily intake of smokeless tobacco. Marketed STPs vary considerably in form and content of toxicants, including nicotine, and thereby in associated health effects, such as increased heart rate, an increased risk in pregnancy, increased premature fetal death, and SIDS (Sudden Infant Death Syndrome). It has been reported by the Scientific Committee on Emerging and Newly Identified Health Risks (SCENIHR) in 2008[11] that aqueous and organic extracts of American and Swedish moist snuff and Indian chewing tobacco cause mutations and chromosomal damage in bacterial and mammalian cell cultures. Increased micronuclei formation in oral epithelial cells as evidence of chromosomal damage in oral cancer patients due to STP use has been reported[12,13].

The role of copper (Cu) in submucous fibrosis *in vitro* has been shown earlier[14] and it was noticed that Cu in gutkha may be responsible for the fibrosis in mouth cavities. Early symptoms of chronic Cu poisoning include precancerous oral lesions (leukoplakia-small white patches) and sores in the mouth or tongue, followed by oral submucous fibrosis and difficulty in opening the mouth fully. Lead is particularly dangerous for the younger age group, as chronic exposure resulting in the lowering of the IQ and its poisoning effect on the brain may not be reversible[4,15,16]. Arsenic exposure can cause skin pigmentation and cancer problems, ulcerations of the mouth, low hemoglobin, leukemia, acute renal failure, seizures, and nerve damage[17], and it is also a potential carcinogen[18]. Excessive doses of Cd are known to cause lung and bone damage, and increased blood pressure[3] and causation of cardiovascular disease[19,20,21].

Our study targeted seven different groups of STPs for determination of a total of eight metals, namely Pb, As, Cu, Se, Cd, Ni, chromium (Cr), and Hg. On analysis, it was found that the smokeless tobacco contained considerable levels of metals like Pb, As, Cd, and Cu, which if consumed in excess would prove harmful for consumers.

## METHODOLOGY

### Sample Collection

Indian STPs were purchased from retail stores in Mumbai. The date of purchase, name of manufacturer, company, and manufacturing date were recorded. Twenty-five forms of different brands of STPs were collected from 10 outlets in various parts of the city. Samples of the same brand were mixed together to obtain a representative sample of that product. Brand names have not been disclosed in this paper due to legal requirements. The products collected for analysis represented the commonly used brands, such as the popular gutkha, zarda, khaini, and mishri, which are chewing tobacco products that have become more popular, especially among teenagers and young adults in many states of India. Other tobacco-containing products were also selected that included creamy snuff and Dentifrice (Dantmanjan). One earlier pharmacokinetic study on mishri (a form of STP) use by women reported that 0.5–1 g of mishri was applied to teeth and retained in the mouth for 10–15 min or longer[22].

### Sample Pretreatment

All samples were ground to 20-mesh size with a grinder and kept in cleaned polythene bags and stored in desiccators. One gram of sample was taken for specific analysis and pretreated as per the standard procedure given below for different metals.

A. Pb, Cd, As, Se, Ni — Wet acid digestion method: a known quantity of sample (1 g) was taken in a clean borosilicate beaker. The sample was wet digested with 3 ml of concentrated nitric acid (AR or GR grade) and 2 ml perchloric acid by slow heating. The solution was evaporated to near dryness. It was ensured that the residue obtained after digestion was free from organic matter, which otherwise act as impurities in metal analysis. The white residue was reconstituted using 10 ml of supporting electrolyte. This sample was further analyzed on the voltammeter, an instrument known for its sensitivity up to nanogram level. This routine analytical method is well known for its minimum detection level for samples having a low concentration of the analyte.

B. Hg — Bethge's apparatus method: 1 g of ground sample, 4 ml of $HNO_3$, and 2 ml of $H_2O_2$ was kept for refluxing for 2 h. This was followed by addition of supporting electrolyte. The sample was then taken for analysis in a cold vapor Hg analyzer.

## Sample Analysis

Levels of Pb, Cd, Cr, Ni, Se, and As in the processed samples were estimated by the differential pulse anodic stripping voltammetry (DPASV) technique, and As was estimated by square wave voltammetry using a EG&G (Princeton, NJ) model PARC 394 with static mercury drop electrode (SMDE) assembly (PARC 303) with a PARC 305 stirrer. The measurement procedure was followed as reported earlier[23]. The electrolytes and instrument conditions used for different metals have been summarized in Table 1. Reagent blank samples were subtracted from the corresponding batch of field samples. The minimum detection limit achieved was 0.03, 0.012, 0.01, 0.02, 0.005, 0.0002, and 0.1 ppm for Pb, Cu, Cd, Cr, Ni, Se, and As, respectively. The quality of data was assured by analysis of standard reference materials[24,25]. The recovery was found to be within ±8%. The permissible levels of metals are summarized in Table 2. Mercury metal in the processed tobacco samples was analyzed using a Digital Mercury Analyzer (MA 5840) from Electronic Corporation of India (ECI), employing a cold vapor generating system using the protocol described in our earlier studies[26].

**TABLE 1**
**Instrumental Parameters Employed for Analysis of Heavy Metals during Voltammetry**

| Metal | E ½ vs. Ag/AgCl | Scan Rate | Pulse Repetition Time | Modulation Amplitude | Supporting Electrolyte Composition | Technique |
|---|---|---|---|---|---|---|
| Pb<br>Cd<br>Cu | –0.42 V<br>–0.6 V<br>–0.02 V | 5 mV/sec | 0.5 sec | 50 mV | 0.25% $HNO_3$ | DPASV |
| Cr | –1.2 V | 5 mV/sec | 0.5 sec | 50 mV | Sodium acetate (0.2 mol/l), diethylenetriamine pentaacetic acid (0.05 mol/l), sodium nitrite (2.5 mol/l) | DPASV |
| Ni | –-0.9 V | 2 mV/sec | 0.5 sec | 50 mV | Double distilled water, 0.02 *M* dimethylglyoxime, ammonia buffer | DPASV |
| Se | –0.6 V | 2 mV/sec | 0.5 sec | 50 mV | 0.2 *M* HCl | DPASV |
| As | –0.5 V | 2 mV/sec | 0.5 sec | 50 mV | 0.1 *M* HCl | Square wave voltammetry |
| Hg | — | — | — | — | 10% $HNO_3$, stannous chloride | Cold vapor atomic absorption |

During the smokeless tobacco analysis, the products were divided into seven main groups and subgroups depending on the form and brands, respectively. Each value was reported as the mean of triplicate analyses, along with the standard deviation (±) as shown in Table 3.

**TABLE 2**
**Permissible Intake Levels as per FAO/WHO Recommendations**

| Metal | Provisional Tolerable Weekly Intake (µg/kg/week) | Per Day Intake (µg/kg/day) | For a 60-kg Individual (µg/day) | Ref. |
|---|---|---|---|---|
| Pb | 25 | 5 | 300 | FAO/WHO |
| As | 15 | 3 | 180 | FAO/WHO |
| Cd | 3.5 | 0.2–1 | 30 | WHO/JECFA |
| Cu | 500 | 100 | 600 | FAO/WHO |

## RESULTS AND DISCUSSION

In India, the consumption of smokeless tobacco is widespread because it is inexpensive, widely available, heavily advertised, and has complete social acceptance, unlike smoking. Nicotine content, toxicants like TSNAs, PAHs, alkaloids, aldehydes, and more have been studied by many[27], so in this study we targeted the determination of metals in the STPs. Recent studies have reported levels of toxic metals in STPs such as snuff and Alaskan iqmik[28], but the research on metals in Indian smokeless tobacco brands is uncommon[7]. Similarly, the use of voltammetry for metals in cigarette tobaccos has been reported earlier[29]. Dobrowolski and Mierzwa reported a comparative analysis of the metals in tobacco using two different techniques involving atomic absorption spectrophotometry[30].

The metal analysis of smokeless tobacco has yielded very useful information about the indirect intake of heavy metals. In these products, the levels of Pb, As, Cd, and Cu exceeded the average daily intake values of consumption. Exposures to each of these were calculated using an average consumption of 10 pouches per day based on survey reports. According to the GYTS (Global Youth Tobacco Surveys) and NSS (National Service Scheme) Unit of TISS (Tata Institute of Social Sciences) Mumbai India 1998, the survey shows that among students, addiction to the following forms of tobacco intake was cigarettes (smoking), 10.6%; tobacco chewing, 6.7%; paan masala, 9.9%; and gutkha, 9.6%. Of those who took these products, very few were addicted to a single product — 15% of those who smoked, 2% of those who ate paan masala, 13% of those who ate gutkha, and 14% of those who chewed tobacco in other forms. Paan masala/gutkha addiction was found in both rural and urban areas[31]. The average daily intake was calculated as shown below.

Daily intake (µg/day) = Metal concentration in gutkha sample taken for analysis × Weight of gutkha sample taken for analysis (pouch weight) × Daily intake of pouch (10 pouches per day)

Several food products in India have recommended limits for some heavy metals; however, although STPs are classified under foods for regulatory purposes, the limits for metals have not been specified. Therefore, the daily intake of these elements was compared with the provisional tolerable weekly intake (PTWI) and the proposed maximum permissible level suggested by the Food and Agriculture Organization /World Health Organization (FAO/WHO), as shown in Table 2.

- **Lead** — The FAO/WHO established a PTWI of Pb in adults and children as 25 µg/kg/week[32,33]. According to the Joint FAO/WHO Expert Committee on Food Additives (JECFA)[33], the accumulation of Pb in the body was based on net absorption of Pb — 40% from dietary sources, 10% from food and drinking water, and up to 50% from inhalation of Pb compounds. This implies that at an intake of 5 µg/kg bw/day, retention of Pb in the body leads to an increased blood Pb level, thereby impacting the hematic and immune system. It was found that in four brands, A5, A7, A8, A9 from group A (gutkha), the Pb level touched or exceeded this permissible range, as shown in Table 3, when an average 10 pouches were consumed per day.

**TABLE 3**
**Observed Levels of Heavy Metals in STPs with (±) Standard Deviation**

| Smokeless Tobacco Forms | Pb | | As | | Cd | | Cu | |
|---|---|---|---|---|---|---|---|---|
| | µg/g | µg/day* | µg/g | µg/day* | µg/g | µg/day* | µg/g | µg/day* |
| A. Gutkha | | | | | | | | |
| A1 | 14.9 ± 0.01 | 149.0 | 0.11 ± 0.01 | 1.1 | 0.13 ± 0.01 | 1.3 | 30.5 ± 0.02 | 305.0 |
| A2 | 0.13 ± 0.02 | 1.3 | 0.57 ± 0.04 | 5.7 | 0.17 ± 0.00 | 1.7 | 36.1 ± 0.02 | 361.0 |
| A3 | 0.25 ± 0.01 | 2.5 | 1.09 ± 0.02 | 109.0 | 0.08 ± 0.00 | 0.8 | 14.7 ± 0.02 | 146.0 |
| A4 | 0.03 ± 0.01 | 0.3 | 0.13 ± 0.01 | 1.3 | 0.01 ± 0.01 | 0.1 | 14.7 ± 0.02 | 147.0 |
| A5 | 68 ± 0.03 | 680.0 | 0.13 ± 0.01 | 1.3 | 3.2 ± 0.02 | 32.0 | 282 ± 0.02 | 2820.0 |
| A6 | 0.95 ± 0.02 | 9.5 | 3.5 ± 0.02 | 35.0 | 0.01 ± 0.01 | 0.1 | 17 ± 0.02 | 170.0 |
| A7 | 29.8 ± 0.04 | 298.0 | 0.12 ± 0.01 | 1.2 | 0.01 ± 0.01 | 0.1 | 272 ± 0.02 | 2720.0 |
| A8 | 23.8 ± 0.02 | 238.0 | 0.13 ± 0.01 | 1.3 | 0.01 ± 0.01 | 0.1 | 237 ± 0.02 | 2370.0 |
| A9 | 33.3 ± 0.01 | 333.0 | 0.14 ± 0.01 | 1.4 | 0.01 ± 0.01 | 0.1 | 656 ± 0.02 | 6560.0 |
| A10 | 0.03 ± 0.01 | 0.3 | 0.34 ± 0.05 | 3.4 | 0.19 ± 0.00 | 1.9 | 14.7 ± 0.02 | 147.0 |
| A11 | 0.15 ± 0.00 | 1.5 | 0.68 ± 0.05 | 6.8 | 0.07 ± 0.00 | 0.7 | 14.7 ± 0.02 | 14.70 |
| A12 | 0.19 ± 0.01 | 1.9 | 0.26 ± 0.05 | 2.6 | 0.11 ± 0.01 | 1.1 | 25 ± 0.02 | 250.0 |
| A13 | 0.15 ± 0.00 | 1.5 | 0.14 ± 0.01 | 1.4 | 0.01 ± 0.01 | 0.1 | 17 ± 0.02 | 170.0 |
| B. Zarda | | | | | | | | |
| B1 | 0.53 ± 0.00 | 5.3 | 0.37 ± 0.02 | 3.7 | 0.23 ± 0.00 | 2.3 | 17.7 ± 0.02 | 177.0 |
| B2 | 0.96 ± 0.03 | 9.6 | 0.39 ± 0.04 | 3.9 | 0.50 ± 0.00 | 5.0 | 18.7 ± 0.02 | 187.0 |
| C. Creamy Snuff | | | | | | | | |
| C1 | 0.13 ± 0.00 | 1.3 | 0.79 ± 0.07 | 7.9 | 0.07 ± 0.00 | 0.7 | 7.7 ± 0.02 | 77.0 |
| C2 | 0.59 ± 0.01 | 5.9 | 0.6 ± 0.07 | 6.0 | 0.15 ± 0.01 | 1.5 | 7.7 ± 0.02 | 77.0 |
| D. Dentifrice (Dantmanjan) | | | | | | | | |
| D1 | 5.04 ± 0.07 | 50.4 | 0.2 ± 0.07 | 2.0 | 0.23 ± 0.02 | 2.3 | 7.7 ± 0.02 | 77.0 |
| D2 | 4.91 ± 0.07 | 49.1 | 0.13 ± 0.04 | 1.3 | 0.18 ± 0.01 | 1.8 | 4.7 ± 0.02 | 47.0 |
| E. Khaini | | | | | | | | |
| E1 | 0.33 ± 0.03 | 3.3 | 0.14 ± 0.02 | 1.4 | 0.04 ± 0.00 | 0.4 | .012 ± 0.02 | 0.12 |
| E2 | 0.31 ± 0.03 | 3.1 | 0.11 ± 0.03 | 1.1 | 0.03 ± 0.01 | 0.3 | .013 ± 0.01 | 0.13 |
| F. Mishri | | | | | | | | |
| F1 | 0.30 ± 0.01 | 3.0 | 0.81 ± 1.00 | 8.1 | 0.11 ± 0.00 | 1.1 | 8.7 ± 0.02 | 87.0 |
| F2 | 0.21 ± 0.01 | 2.1 | 0.79 ± 0.02 | 7.9 | 0.10 ± 0.01 | 1.0 | 8.2 ± 0.02 | 82.0 |
| G. Others | | | | | | | | |
| G1 | 0.16 ± 0.02 | 1.6 | 0.83 ± 0.07 | 8.3 | 0.07 ± 0.00 | 0.7 | 8.7 ± 0.02 | 87.0 |
| G2 | 0.16 ± 0.02 | 1.6 | 0.82 ± 0.07 | 8.2 | 3.1 ± 0.01 | 31.0 | 8.6 ± 0.01 | 86.0 |

* Per day levels calculated considering a consumption of minimum 10 pouches per day.

- **Arsenic** — In 1990, the JECFA set the As level to 2.1 µg/kg bw/day. PTWI for As according to the FAO/WHO in adults is 15 µg/kg/week[32,34,35,36]. According to this level, brand A3 from group A was found to contain toxic levels of As, as shown in Table 3.
- **Cadmium** — Cd was found below the permissible level in all groups, as shown in Table 3, except from Group A and G, brands A5 and G2, respectively. According to WHO-JECFA[33,37], the recommended value of Cd is 3.5 µg/kg bw/week for adults. Considering the accumulation property and the long biological half-life of Cd, a level of 0.2–1 µg/kg bw/day has been set[32,37]. This equals 30 µg Cd/day for a 60-kg body weight individual. The absorption following oral exposure of Cd is likely to depend on physiological status, such as age and levels of Fe, Ca, and Zn stored in the body. According to IPCS 1992[19], ingested Cd from daily food and water is about 12–25 µg, from which the actual absorbed amount of Cd is 0.6–1.3 µg/day, and total inhalatory intake from the atmosphere is 0.15 µg/day of which the actual absorbed amount of Cd is 0.04 µg/day.

- **Copper** — Cu is an essential and beneficial element in human metabolism. Its recommended daily intake, based on essentiality, is about 0.5 mg/kg bw/day, i.e., 500 µg/kg body weight per day according to FAO/WHO[38,39]. The Cu levels were found to be higher in four brands: A5, A7, A8, and A9 of Group A — 282, 272, 237, and 656 µg per pouch as shown in Table 2. As per earlier studies, the average daily exposure from air, food, and water for a person weighing 70 kg and drinking 1.5 l of water per day, eating 1.5 kg of food per day, and inhaling 20 $m^3$/day is 0.01–0.06 µg/kg, 31.4 µg/kg, and 3.77 µg/kg body weight per day, respectively[40]. The role of Cu in gutkha for causing submucous fibrosis has been of concern among dentists.

The metals Ni, Se, Hg, and Cr were found to be negligible, and therefore not reported in the results. The source of heavy metals in smokeless tobacco may be due to atmospheric absorption by tobacco plants[41]. A number of factors influence the actual level of elements found in plants that include type of plant tissue, level of elements in soil, soil and leaf residues resulting from application of metal-containing pesticides, insecticides[42], and soil amendments including fertilizers and municipal sludge[43]. It also depends on the distance of the plant from the source of the element, the season, the climatic condition, and the foliar uptake from settled aerosols[6]. Also, it is possible that the source of metals may be due to the addition of various ingredients as shown in Table 4. Certain spices, such as mint, saffron, etc., used in the flavoring of STPs might also contribute to the heavy metal content[44].

**TABLE 4**
**Common Ingredients Used for Flavoring of Tobacco**

| S. No. | Name | Ingredients |
|---|---|---|
| 1 | Gutkha | Areca nut, catechu, tobacco, lime, saffron, flavoring agent, saccharine, mint. Held in the mouth and chewed. |
| 2 | Zarda | Loose leaves boiled in water with lime and spices to evaporation; the residual particles are then dried and colored with vegetable dye. |
| 3 | Creamy Snuff | Fire cured tobacco; after the initial curing process, the leaves undergo fermentation process, then are enriched with flavor additives, including spices. |
| 4 | Tooth Powder | Tobacco, clove oil, glycerin, menthol, spearmint, camphor. |
| 5 | Khaini | Mixture of tobacco, lime, menthol, or aromatic spices. |
| 6 | Mishri | Powdered form of roasted tobacco. |
| 7 | Other tobacco | Mixture of tobacco, lime, menthol, or spices. |

To summarize, the gutkha brands tested had significant levels of metals as compared to other groups of smokeless tobacco in the range: Pb (0.03–68 µg/g), Cd (0.01–3.2 µg/g), As (0.1–3.5 µg/g), and Cu (0.012–656 µg/g). This study emphasizes the fact that some STPs have heavy metals above permissible limits prescribed by the WHO. This study was also instrumental for the policy makers in order to impose a temporary ban on the sale of the popular brand gutkha in Maharashtra, a state in India.

## ACKNOWLEDGMENTS

We thank the Department of Scientific and Industrial Research (DSIR), Government of India for granting us a Scientific and Industrial Research Organization (SIRO) status, thereby promoting our research endeavors. Our parent organization, Foundation for Environmental Medicine (FEM) Mumbai, provided us funds for this research.

## References

1. Atrens, D.M**.** (2001) Nicotine is an additive substance: a critical examination of the basic concepts and the empirical evidence. *J. Drug Issues*. **32**, 325–394.
2. IARC Monographs (1987) *Tobacco Products, Smokeless.* Suppl. 7. International Agency for Research on Cancer: Evaluation of Carcinogenic Risks to Humans. Overall Evaluations of Carcinogenicity: An Updating of IARC Monographs Volumes 1 to 42.
3. IARC Monographs (1993) *Cadmium and Cadmium Compounds.* **Suppl. 58.** International Agency for Research on Cancer: Evaluation of Carcinogenic Risks to Humans.
4. IARC Monographs (2006) *Inorganic and Organic Lead Compounds.* Suppl. 87. International Agency for Research on Cancer: Evaluation of Carcinogenic Risks to Humans.
5. Lars, Jarup. (2003) Hazards of heavy metal contamination. *Br. Med Bull***.** **68**, 167–182.
6. Fergusson, J.E. (1990) *The Heavy Elements: Chemistry, Environmental Impact, and Health Effects.* Pergamon Press, Oxford.
7. Shaikh, A.N., Khandekar, R.N., Anand, J.S., and Mishra, U.C. (1992) Determination of some toxic trace elements in Indian tobacco and its smoke. *J. Radioanal. Nucl. Chem*. **163**, 349–353.
8. Rickert, W.S. and Kaiserman, M.J. (1994) Level of lead, cadmium, and mercury in Canadian cigarette tobacco as indicators of environmental change: result from a 21 year study. *Environ. Sci. Technol*. **28**, 924–927.
9. Golia, E.E., Dimirkou, A., and Mitsios, I.K. (2007). Accumulation of metal on tobacco leaves (primings) grown in agriculture area in relation to soil. *Bull. Environ. Contam. Toxicol*. **79**, 158–162.
10. Zhang, Y. (2004) Summary on study of heavy metal elements in tobacco. *Tobacco Sci. Technol*. **27**, 20–23.
11. SCENIHR (2008) Health Effect of Smokeless Tobacco Products. Scientific Committee on Emerging and Newly Identified Health Risks. European Commission, Brussels.
12. Rodu, B. and Jansson, C. (2004) Smokeless tobacco and oral cancer: a review of the risk and determinants. *Crit. Rev. Oral Biol. Med*. **15**, 225–263.
13. Rodu, B. and Cole, P. (2005) Smokeless tobacco and periodontal disease. *J. Dent. Res.* **84**, 1086–1087.
14. Trivedy, C., Meghji, S., Warnakulasuriya, K.A.A.S., Johnson, N.W., and Harris, M. (2001) Copper stimulates human oral fibroblasts in vitro: a role in the pathogenesis of oral sub mucous fibrosis. *J. Oral Pathol. Med*. **30**, 465–470.
15. Raghunath, R., Tripathi, R.M., Khandekar, R.N., and Nambi, K.S.V. (1997) Retention times of Pb, Cd, Cu, Cu and Zn in children’s blood. *Sci. Total Environ*. **207**, 133–139.
16. Khandekar, R.N., Mishra, U.C., and Vohra, K.G. (1984) Environmental lead exposure of urban Indian population. *Sci. Total Environ*. **40**, 269–278.
17. Byrd, D.M., Roegner, M.L., Griffiths, J.C., Lamm, S.H., Grumski, K.S., Wilson, R., and Lai, S. (1996) Carcinogenic risk of inorganic arsenic in perspective. *Int. Arch. Occup. Environ. Health* **68**, 484–494.
18. La Grega, M.D., Buckingha, P.L., and Evens, J.C. (1994) *Hazardous Waste Management.* McGraw-Hill, New York.
19. IPCS (1992) International Programme on Chemical Safety: Environmental Health Criteria: Cadmium. Ser. 134. World Health Organization, Geneva.
20. ATSDR (1997) Agency for Toxic Substances and Diseases Registry. Toxicological Profile for Cadmium. Draft for public comment. Department of Health and Human Services, Public Health Service, Atlanta.
21. Jarup, L., Berglund, M., Elinder, C.H., Nordberg, G., and Vahter, M. (1998) Health effects of cadmium exposure and risk estimate. *Scand. J. Work Environ. Health* **24**, 1–52.
22. Gupta, C.P. and Sreevidya, S. (2004) Smokeless tobacco use, birth weight, and gestational age: population based prospective cohort study of 1217 women in Mumbai, India. *Br. Med. J*. **328**, 1538–1540.
23. Khandekar, R.N., Dhaneshwar, R.G., Palrecha, M.M., and Zarapkar, L.R. (1981) Simultaneous determination of lead, cadmium and zinc in aerosols by anodic stripping voltammetry. *Fresen. J. Anal, Chem*. **305**, 365–368.
24. Tripathi, R.M., Khandekar, R.N., Raghunath, R., and Mishra, U.C. (1989) Assessment of atmospheric pollution from two heavy metals in two cities of India. *Atmos. Environ*. **23**, 879–883.
25. Khandekar, R.N., Tripathi, R.M., Raghunath, R., Sathe, A.P., and Nambi, S.V. (1992) Heavy metal concentration in air, water and rock samples at Antarctica during 1989-1990. *Curr. Sci. India* **63**, 201–204.
26. Deshpande, A., Bhendegiri, S., Shirsekar, T., Dhaware, D., and Khandekar, R.N. (2008) Analysis of heavy metals in marine fish from Mumbai docks. *Environ. Monit. Assess*. [Epub ahead of print]

27. Stepanov, I., Hecht, S.S., Ramakrishna, S., and Gupta, P.C. (2005) Tobacco-specific nitrosamine in smokeless tobacco products marketed in India. *Int. J. Cancer* **116**, 16–19.
28. Pappas, R.S., Stenfil, S.B., Watson, C.H., and Ashley, D.L (2008) Analysis of toxic metals in commercial moist snuff and Alaskan iqmik. *J. Anal Toxicol.* **32,** 281–291.
29. Recai, S. and Hasan, A. (1996) Determination of lead, copper and selenium in Turkish and American cigarette tobaccos by anodic stripping voltammetry. *Anal. Sci*. **12,** 911–915.
30. Dobrowolski, R. and Mierzwa, J. (1992) Direct solid vs slurry analysis of tobacco leaves for some trace metals by graphite furnace AAS: a comparative study. *Fresen. J. Anal. Chem.* **334,** 340–344.
31. Ray, C.S. (2003) Research on Tobacco in India (Including Betel Quid and Areca Nut). An Annotated Bibliography of Research on Use, Health Effects, Economics, and Control Efforts. World Bank, Washington, D.C.
32. Baars, R.J., Theelen, R.M.C., Janssen, P.J.C.M., Hesse, J.M., Apeldoorn, M.E., Van Meijerink, M.C.M., Verdam, L., and Zeijmaker, M.J. (2001) Re-Evaluation of Human-Toxicological Maximum Permissible Risk Levels. RIVM Report 711701 025. National Institute for Public Health and the Environment, Bilthoven, The Netherlands.
33. FAO/WHO (1993) Expert Committee on Food Additives: Evaluation of Certain Food Additives and Contaminants. 41st Meeting of the Joint FAO/WHO. Ser. 837. World Health Organization, Geneva.
34. Seiler, H., Sigel, A., and Sigel, H. (1994) *Handbook on Metals in Clinical and Analytical Chemistry*. Marcel Dekker, New York.
35. Delgado-Andrade, C., Navarro, M., Lopez, H., and Lopez, M.C. (2003) Determination of total arsenic levels by hydride generation atomic absorption spectrometry in foods from south-east Spain: estimation of daily dietary intake. *Food Addit. Contam*. **20**, 923–932.
36. Qupirolo, F., Stcgen, S., Restovic, M., Paz, M., Ostapczuk, P., Schwuger, M.J., and Munoz, L. (2000) Total arsenic, lead, and cadmium levels in vegetables cultivated at the Andean villages of northern Chile. *Sci. Total Environ*. **255**, 375–384.
37. FAO/WHO (1989) Expert Committee on Food Additives: Toxicological Evaluation of Certain Food Additives and Contaminants. 33rd Meeting of the Joint FAO/WHO. Ser. 24. World Health Organization, Geneva.
38. FAO/WHO (1970) Expert Committee on Food Additives: Toxicological Evaluation of Some Extraction Solvents and Certain Other Substances. 14th Meeting of the Joint FAO/WHO. Ser. 48A**.** World Health Organization, Geneva.
39. WHO (1996; 1998) Guidelines for Drinking Water Quality. 2nd ed. In Health Criteria and Other Supporting Information. World Health Organization. Geneva.
40. DSP (1979) Depository Service Programme of Canada. Collection. Copper - Canadian Exposure. Updated February 1992.
41. Asta, J., Guillard, E., Tissut, M., Gaude, T., and Ravanel, P. (2003) Heavy metal transfer from atmosphere to plants. *J. Phys*. **107**, 65–67.
42. WHO (2000) Arsenic**.** Air Quality Guidelines. 2nd ed. WHO Regional Office for Europe, Copenhagen.
43. Lugon-Moulin, N., Ryan, L., Donini, P., and Rossi, L. (2006) Cadmium content of phosphate fertilizers used for tobacco production. *Agron. Sustain. Dev*. **26**, 151–155.
44. Divrikli, U., Horzum, N., Soylak, M., and Elci, L. (2006) Trace heavy metal contents of some spices and herbal plants from western Anatolia, Turkey. *Int. J. Food Sci. Technol*. **41**, 712–716.
